# Placenta Previa*Read before Louisville Clinical Society.

**Published:** 1895-03

**Authors:** P. Guntermann

**Affiliations:** Louisville, Ky.


### PLACENTA PREVIA.*

BY P. GUNTERMANN, M.D.,
LOUISVILLE, KY.

Placenta previa is said to occur about once in five hundred or seven hundred and thirty-three pregnancies.

*Cause.*—Since placenta previa is mostly found in multipara and in women who have suffered with leucorrhea, it is supposed that a large uterine cavity with a smooth surface deprived of its fold and epithelium has much to do with the aberration. Recently it has been broached, and the theory is plausible, that the impregnated

*Read before Louisville Clinical Society.

ovum had become detached from its usual seat one of the cornu of the uterus and, instead of being lost or absorbed, had lodged near the internal os and there developed.

The placenta may be placed centrally or laterally. In placenta centralis hemorrhage is of necessity larger and more dangerous than in placenta lateralis; in the latter instance a woman may go on to full term and even be delivered without the loss of more blood than ordinary. The cause of the hemorrhage is due to rupture of the blood-vessels, and comes mostly from the placental vessels. We seldom have hemorrhages from placenta previa during the first six months of gestation, nearly always during the last three months, certainly at the onset and progress of labor. The reason for this, we are taught, is that during the first two-thirds of pregnancy the upper two-thirds of the uterus enlarge, and in the last three months the fibers of the lower segment multiply and grow rapidly. Now, also, the placenta has fully developed within the first six months, and, if placed in the lower segment over or at the side of the os internum, as the inferior third of the uterus begins to enlarge and expand, it (the placenta) remains passive, does not at least grow in proportion to the uterine structure. The necessary consequence is detachment, rupture of vessels, and bleeding.

*Diagnosis.*—Sudden, free, and unaccounted-for hemorrhage from the vagina in a pregnant woman is the first and almost positive diagnostic sign of placenta previa. Palpation reveals an unusually globular uterus; it is not pyriform or even ovular. The child may be readily felt through the thin walls at the fundus. On auscultation we detect the placental bruit over the hypogastric region. The os and cervical region has a peculiar boggy feel. When the os is open, the placenta central, an educated finger will at once detect the placental structure and, when lateral, the membranes are abnormally thick and lead to the placental margin.

*Prognosis.*—As may be inferred from the preceding the prognosis is very grave. From ten per cent. to forty per cent. of the mothers die. When the mother does not perish, she is liable to be an invalid for a long and indefinite time; she is exceedingly prone to septic infection. The death-rate of the children is simply appalling. Statistics give it as from fifty per cent. to seventy-five per cent.

*Treatment.*—In the early period of pregnancy all hemorrhages from the vagina must be treated on the expectant plan, the more so if we are not quite certain as to their cause. We enjoin rest—absolute quiet of body and mind and the recumbent posture. In the latter months with the os still indurated and enlarged, the same rule holds good; supplementary we may insert a tampon. A good cotton or charpie tampon is preferable to a C. Braun's colpeurynter or any other artificial contrivance. The tampon will check hemorrhage, at least in a measure, and superinduce labor pains.

As soon as the os softens and opens to allow the passage of a finger, commence to dilate gradually but surely. When dilatation has sufficiently progressed—this to be determined by the operator—turn by the bimanual or Braxton Hicks method, detach the placenta and pull one or both of the lower limbs down. Here we have a tampon, the size of which may be increased by slowly pulling the limb or limbs. Detach the placenta on the side where it has the least volume; if central, detach on the side where the feet of the baby are felt. It is perhaps never advisable or safe to pass directly through a central placenta because:

First. We necessarily tear large blood-vessels and may cause fatal hemorrhage.

Second. It is by no means easy to go through such a large fleshy mass, and a separation of a more or less large area of placenta from the uterine walls is unavoidable.

Third. The central opening may be too small to allow the speedy passage of the child's body, or, if it does, it may disturb the proper position of the limbs or distend the head, making delivery tedious and enhance the danger from prolonged hemorrhage. Sometimes it may be well to rupture the membranes. The contracting uterus closes the sinuses, forces the body, or more desirable, the head of the child against and upon the bleeding surface, and thus the hemorrhage is checked. This, however, is a dangerous procedure and may become the cause of fatal concealed hemorrhage. The bleeding may cease spontaneously. When the child is dead, the blood frequently coagulates in the sinuses and seals the bleeding vessels.

Dilatation is often accomplished by artificial means. Never use a sponge tent; it increases the danger of septic infection. Barnes's dilators are universally used. They ought to be inflated with air or water. Air is the only agent that will dilate them to their fullest extent; water oftentimes bursts them. These dilators act also the part of a tampon.

An *accouchement forcé* is not devoid of peril; yes, it is at times disastrous. Should the neck not be fully obliterated, softened, and dilatable, extensive lacerations are almost unavoidable, and from them abundant hemorrhages flow.

Our guide for rapid or slow delivery must be the condition of the child. If the child is dead we can temporize; on the other hand, if it is alive, it is our duty to save it by quick and forcible extraction. Turn or use the forceps, as the case may be, and do it promptly. Altogether, each case must be treated on its own merits, and the management must be left to the discretion and ability of the attendant.

Medicines are of little value. Quinine is an excellent oxytocic and uterine tonic; so is strychnine, which is also one of our best cardiac stimulants. Injections of whisky or ether may be called for to sustain the ebbing life until the crisis is passed. Astringents are useless. The utility of ergot—preferably given hypodermatically—is questionable; so are local applications. Yet ergot, hot and cold applications, either singly or combined, and astringents may have a large share in the treatment.

The rule in the management of placenta previa should be: Safety to the mother first and then to the child.

